# Mental Workload and Job Satisfaction in Healthcare Workers: The Moderating Role of Job Control

**DOI:** 10.3389/fpubh.2021.683388

**Published:** 2021-09-03

**Authors:** Fatemeh Rostami, Amin Babaei-Pouya, Gholamheidar Teimori-Boghsani, Azam Jahangirimehr, Zahra Mehri, Maryam Feiz-Arefi

**Affiliations:** ^1^School of Public Health, Hamadan University of Medical Sciences, Hamadan, Iran; ^2^Department of Occupational Health Engineering, School of Health, Ardabil University of Medical Sciences, Ardabil, Iran; ^3^Department of Occupational Health Engineering, School of Health, Torbat Heydariyeh University of Medical Sciences, Torbat Heydariyeh, Iran; ^4^Department of Public Health, Shoushtar Faculty of Medical Sciences, Shoushtar, Iran; ^5^MSc of Scientometrics, Shoushtar Faculty of Medical Sciences, Shoushtar, Iran; ^6^Department of Occupational Health Engineering, Shoushtar Faculty of Medical Sciences, Shoushtar, Iran

**Keywords:** job satisfaction, job control, mental workload, healthcare worker, hospital

## Abstract

**Objective:** The aim of this study was to investigate the moderating role of job control in relation to mental workload and job satisfaction of healthcare workers.

**Methods:** This cross-sectional study was carried out on 480 nurses, midwives, and administrative workers in four educational hospitals of Ardabil, Iran. Research tools were included demographic information questionnaire, NASA-TLX questionnaire, job description index (JDI) questionnaire and job control inquiry.

**Results:** Compared with administrative workers, mental workload of nurses and midwives was significantly higher and likewise mental workload of nurses was significantly difference compared to midwives (*P* < 0.001). Nurses and midwives had substantially higher job satisfaction than administrative workers (*P* < 0.001). Also, nurses and midwives had higher job control than administrative workers (*P* < 0.001 and *P* = 0.002, respectively). Based on the designed model, the correlation between mental workload and job satisfaction was negative and significant (*r* = −0.22); which in the presence of job control, the relationship between the two variables of workload and job satisfaction slightly increased (*r* = −0.19, *P* < 0.001). These conditions were the same in the three job groups separately.

**Conclusion:** Mental workload is inversely related to job satisfaction and job control. Job control plays an important role in improving working conditions in healthcare workers.

## Introduction

Hospitals are considered as the most hazardous centers for providing services in the health system (1) and healthcare workers, as the caregivers of patients, are exposed to various occupational hazards include exposure to biological, chemicals, physical, safety, and ergonomic and psychosocial agents (2, 3). The outbreak of occupational hazards in healthcare workers has been reported differently (4). According to a national survey in the United State in 2012, the incidence of occupational injuries and illnesses in health employees and medical care was over 6.6%, which is the highest-ranking among 56 service industries (5). Nurses are more at risk than other working groups due to more communication with patients, long and rotating work shifts, and the resulting exhaustion (6) so that the outbreak of occupational hazards has been reported four times more than the other occupations in them (7). Midwifery staff also face risks because of some job problems including a lack of knowledge, stress, high demands in the workplace, on-call, and a lack of management support (8). Studies have shown that psychological factors have a significant impact on the occurrence of complaints; as well as work-related accidents (9, 10). Improving the psychological conditions of the workplace and paying attention to these factors can prevent many difficulties and increase the employees' health (11).

Job satisfaction, as a positive emotional factor, is one of the most important psychological factors related to employees and can motivate them (12, 13). Job satisfaction refers to the attitudes of employees toward their jobs. In other words, it is about satisfying and meeting job needs, workload, and effectiveness (10). It is affected by various factors such as salary, communications, policies, job aspects, work order, and personal features of the employees (14, 15). Therefore, in order to increase the job satisfaction, the improvement of working conditions should be targeted (16). Job dissatisfaction of nurses may lead to absenteeism and poor performance and potentially affects the quality of patient care (17). Studies have revealed a positive correlation between the job satisfaction and job performance of nurses and midwives (18–20).

Mental workload is a complicated and multi-aspect structure that is affected by external work needs, environment, psychological and organizational factors, and mental and organizational abilities (21). Workload, as one of the essential components of service delivery in the health system, has a decisive role in undesirable consequences such as emotional exhaustion, depersonalization, and burnout (22). The results of the study conducted by Paneque and Carvajal showed that 86% of employees are exposed to psychological hazards (23). Work-related stresses, of excessive, can endanger a person's health by causing physical, psychological, and behavioral complications (24). Employees, who do not have a good mental health, will not be able to provide adequate patient care. The mental health of the healthcare workers affects the quality of care provided with patients (25). Therefore, in order to provide a proper care by the employees, it is necessary for them to be healthy people; poor mental health can lead to further occupational difficulties in employees.

Hospital professions are classified into a group of jobs with mental workload because of high workload and different job requirements that may be due to large clientele, high workload, shift work, and lack of employees in healthcare workers (26, 27). The health level of the workers increases by preventing and reducing stressors in hospital workplaces, and consequently, their efficiency and effectiveness increase in providing services (28, 29).

Leiter and Maslach stated that job control plays an important role in burnout and the workload of employees. The job control enables workers to make decisions about their work, and on the other hand, a lack of job control means that employees' authority is limited (30). Portoghese et al. also discussed the moderating role of job control on the relationship between workload and burnout (31). This role is based on demand-job control theory as well as extended models of this theory, such as demand-control-performance. With respect to the demand-job control theory, the stress in work environments occurs when the job demands is high and job control is low (32). Other study showed that job control beyond the job demand has a significant predictive power for burnout (33). Therefore, job control as a potential moderator variable can play a good role in patterns such as demand-control-behavioral consequences. This study was aimed at investigating the level of mental workload and job satisfaction of healthcare workers as well as investigating the moderating role of job control in the correlation between mental workload and job satisfaction of the employees.

## Methods

### Study Design and Subjects

This cross-sectional study was carried out on healthcare workers in four educational hospitals of Ardabil, Iran, in 2021. The healthcare workers were estimated 720. So, 480 numbers of participants considering the inclusion and exclusion criteria were entered into the study. Participants were included nurses (*n* = 188), midwives (*n* = 150), and administrative workers (*n* = 142). Inclusion criteria involved willingness to cooperate and having more than 1 year of work experience in the hospital, and the persons, who were unwilling to cooperate, were excluded from the study. In addition, since the number of physicians participating in the study was very small they were excluded from the study.

**Figure 1:**
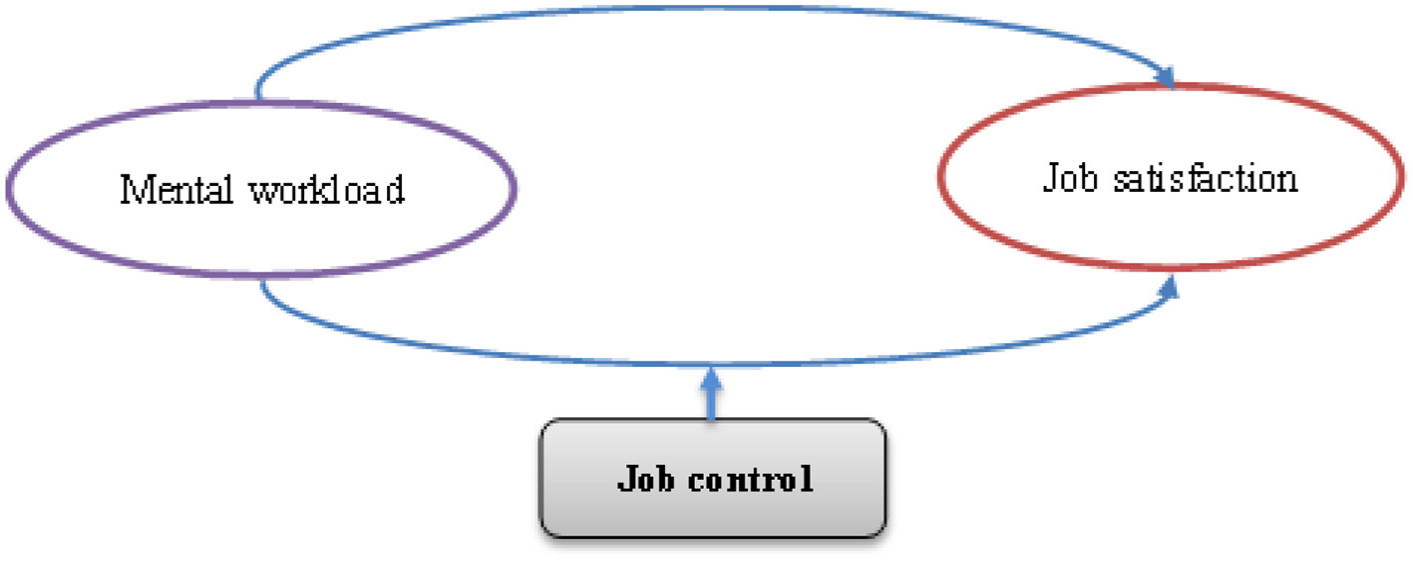
A model of mental workload, the role of job control as a moderating variable, and job satisfaction.

At first, this study was approved by the ethics committee of Shoushtar faculty of Medical Sciences, code no. IR.SHOUSHTAR.REC.1399.043; procedures of this study accorded with principles of declaration of Helsinki (1964) and its later amendments. Then, the necessary coordination was made with the relevant units in the hospitals. The purpose of the study was fully stated for people. The participants were informed about how to fill out the questionnaire along with the administration of the questionnaires. The sampling method was the census and participants expressed their written consent. Data were gleaned using standard questionnaires. Questionnaires including the NASA-TLX questionnaire, job satisfaction questionnaire, and Job Control Assessment (JCA) were used. The demographics information of the participants included age, gender, work experience, education level, primary and second jobs, and type of employment. The hard copies of the questionnaires were distributed to healthcare workers and collected by researchers in 2 weeks. In the present study, the Persian version of the questionnaires was used. In this study, the relationship among workload as an occupational demand and positive behavioral consequences such as job satisfaction, and also job control is considered as the role of a moderating variable, as shown in Figure 1.

### Mental Workload Questionnaire

NASA-TLX questionnaire is one of the workload assessment methods introduced by Hart and Staveland, and it is widely the most powerful tool available for evaluating perceptual aspects of the workload (34). The NASA-TLX is a multidimensional method that provides an overall score of workload based on a weighted average of six scales including six demands such as mental demand, physical, temporal effort, performance, effort, and frustration (35). In this questionnaire, for each field of activity, it is divided into 100 points with 5-point steps. Many studies have confirmed the reliability and validity of this method for assessing workload (36). This questionnaire was translated into Persian by Mohammadi et al. and its validity and reliability were evaluated and Cronbach's alpha coefficient was 0.847. Weighted averages are computed by multiplying the raw score of each scale by the number of times the associated workload factor was chosen in the paired-choice task, then dividing by the sum of the weights (i.e., 15) (37).

### Job Satisfaction Questionnaire

The Job Satisfaction Questionnaire, known as the Job Descriptive Index (JDI), was designed by Smith, Kendall and Huhn at the Cornell University in 1969 (38). This questionnaire is one of the most valid questionnaires used for the evaluation of job satisfaction. It consists of 54 questions and has been used in various studies (16, 39). The Persian version of the questionnaire was measured at Shahid Chamran University of Iran and its validity and reliability coefficients were equal to 0.96 and 0.94, respectively. Its rating scale is five points Likert (1 strongly disagree to 5 strongly agree). The JDI is derived from various aspects of job, such as income, opportunities for promotions, supervisors, colleagues, and work environment factors like supervisors' style, policies and procedures, workgroup affiliation, working conditions, and job benefits (40). The score of each questionnaire is obtained by sum of the scores of questions and total score of job satisfaction is computed by multiplying the score of each questionnaire by the number of questionnaires. The range of score will be 30–150. Job satisfaction status is determined based on the following classification: Score between 30 and 50 Job satisfaction is low, Score between 51 and 102 Job satisfaction is moderate and Score above 102 Job satisfaction is high.

#### Job Control Assessment

The questionnaire of job control (JCA) designed by Adibi et al. contains five questions based on a 5-point Likert scale (1 strongly agree to 5 strongly disagree). The tool measures the job control by quantifying the employees' perceived control over work affairs, methods of work, policies of work, and perceived autonomy during the work. Cronbach's alpha of the five questions was obtained 0.7 Job control score is computed by summation questions score and multiplying the score obtained from each questionnaire with number of questionnaires. The range of job control score is 5–25 (41).

### Statistical Analysis

Data analyses were performed using SPSS 21 and AMOS 18 software. The quantitative variables were reported as mean ± standard deviation and qualitative variables were stated in numbers (percent). The normality of the quantitative variables was assessed using the Kolmogorov-Smirnov test. The chi-square test was used to investigate the relationship between qualitative variables, and one-way analysis of variance was applied to compare the quantitative variables. The *post hoc* tests were performed for pairwise comparison of the quantitative variables. Pearson correlation coefficient was used to determine the relationship between the quantitative variables. Multivariate analysis of variance (MANOVA) was applied for the role of job control. Structural equation models (SEMs) were used to investigate the other variables when exposed to the moderating variable. The significance level was considered to be < 0.05 (*P* < 0.05).

## Results

### Demographic and Job Characteristics

Response rate of participants was 73.2% as 527 forms were received back. Of them, 480 were completely filled and segregated for further analyses. Eighty-nine percent of people were women and 11% were men. The hospital staff surveyed in this study nurses and midwives were 39.2 and 31.2%, respectively, and 29.6% of the participants worked in the official units. Most participants were Bachelor of Science (85.8%), followed by postgraduate (10.0%). The demographic characteristics of the hospital staff are listed in Table 1.

**Table 1:** Demographic variable.

**Variable**	**Categories**		***n*** **(%)**
Job	Nurse		188 (39.2%)
	Midwife		150 (31.2%)
	administrative staff		142 (29.6%)
Education	Associate's degree		20 (4.2%)
	Bachelor		412 (85.8%)
	Master and higher		48 (10.0%)
sex	Male		53 (11%)
	Female		427 (89%)
	**Min**	**Max(years)**	**Mean ± SD**
Age	22	56	34.01 ± 6.02
Job experience	1	29	7.91 ± 7.25

### The Comparison of Mental Workload, Job Satisfaction, and Job Control Scores

The average of mental workload, job satisfaction, and job control scores among all participants was 70.98 ± 15.14, 193.77 ± 55.15, and 14.65 ± 5.65, respectively. The subscales of mental workload (NASA TLX Index) shown in Table 2.

**Table 2:** Mean and standard deviation (SD) Scores obtained for NASA-TLX index.

**NASA TLX index**	**Mean**	**SD**
Physical demand	73.33	23.435
Temporal demand	50.23	26.878
Performance demand	73.84	25.856
Effort demand	75.89	19.905
Frustration demand	77.18	22.102
Mental demand	74.01	27.592
Total score for NASA-TLX	70.9896	15.14901

The relationship between demographic characteristics as age and work experience with the mental workload, job satisfaction and job control shown in the Table 3. The scores of mental workload, job satisfaction, and job control among healthcare workers statistically significant differences among the three groups for job and education (*P* < 0.05). As shown in Table 3. After a further comparison between every two groups by Tukey follow-up test, mental workload of nurses was higher significantly than midwives and administrative workers and also mental workload of midwives was significantly difference compared to administrative workers (*P* < 0.001). Nurses and midwives had substantially higher job satisfaction (*P* < 0.001) and job control than administrative workers (*P* < 0.001 and *P* = 0.002, respectively).

**Table 3:** Mean of underlying variables and their relationship with workload, job satisfaction, and job control.

**Variable**		**Mental workload**		**Job satisfaction**		**Job control**	
		**Mean ± SD**	***P*** **-Value**	**Mean ± SD**	***P*** **-Value**	**Mean ± SD**	***P*** **-Value**
Job	Nurse	77.69 ± 12.13	<0.001^*^, *F* = 56.59	185.31 ± 54.86	<0.001^*^, *F* = 9.81	14.38 ± 5.81	NS
	Midwife	71.50 ± 13.16		188.49 ± 54.94		14.47 ± 5.82	
	administrative staff	61.57 ± 15.86		210.54 ± 52.34		15.19 ± 5.48	
Education	Associate's degree	73,055 ± 8.87	0.714, *F* = 0.336	134.05 ± 34.29	<0.001^*^, *F* = 13.70	12.55 ± 6.00	NS
	Bachelor	70/81 ± 15.55		195.27 ± 25.83		14.71 ± 5.75	
	Master and higher	71.45 ± 13.72		205.75 ± 39.63		14.97 ± 5.20	
**Variable**				***r***, ***P*****-value**			
Age		0.235, <0.001^*^		−0.894, <0.001^*^		−0.051, NS	
Job experience		0.224, <0.001^*^		−0.683, <0.001^*^		−0.048, NS	

* *significance level at p = 0.001*.

### The Model Results

**Figure 2:**
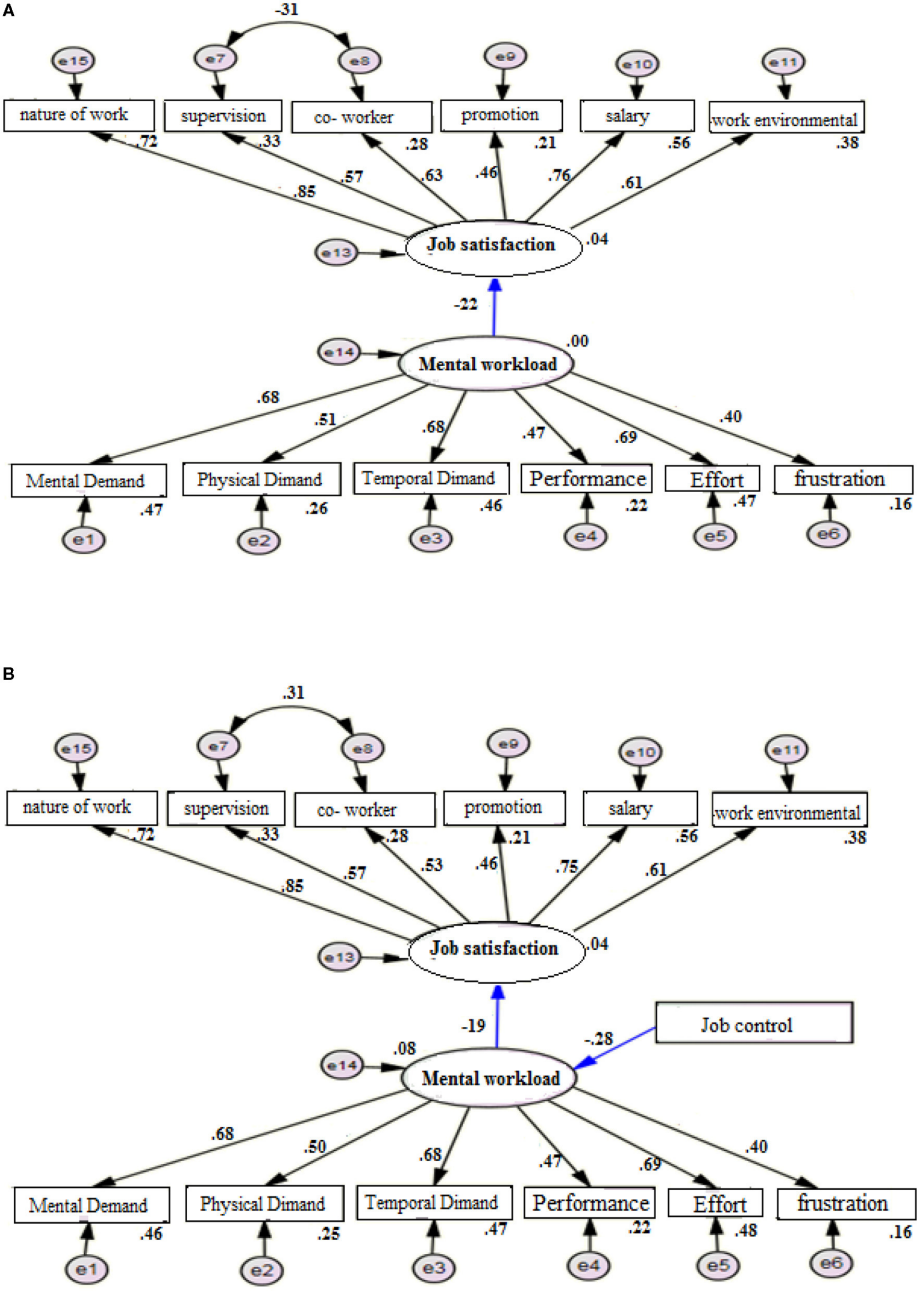
**(A)** The model of relationship between job satisfaction and mental workload without job control moderating variables. **(B)** The model of relationship between job satisfaction and workload with moderating variable of job control.

Figure 2, designed as model 1a shows the correlation between workload and job satisfaction (*r* = −0.22, *P* < 0.001). According to this model, job satisfaction decreased by increasing the workload. Based on the results in Table 4, some goodness of fit indices showed that the designed model has a good fit. In the designed model 1b, the role of job control as mediating variable was plotted. The standard coefficient between the workload and job satisfaction was *r* = −0.19, *P* < 0.001, and the standard coefficient of job control on subjective workload was *r* = −0.28, *P* < 0.001. That is, in the presence of the job control (which has a significant and inverse relationship with the mental workload), the relationship between the two variables of workload and job satisfaction increases slightly. Some goodness of fit indices of the designed model for investigating the correlation between workload and job satisfaction despite the job control showed that the designed model is well-fitted (Table 4). According to the results, among the dimensions of mental workload, the highest score was related to effort followed by the pressure of time, and stress, respectively. Among the aspects of job satisfaction variable, the highest score was related to the job nature followed by income and the work environment (Figure 2).

**Table 4:** Goodness criteria of model fit in relationship between mental workload, job satisfaction, and moderating variable of job control.

**Goodness criteria of model fit**	**CFI^a^**	**RMSEA^b^**	**TLI^c^**	**CMIN/DF^d^**	**IFI^e^**
Without the job control	0.916	0.071	0.893	3.435	0.916
Presence of job control	0.918	0.64	0.899	2.985	0.919
Acceptable level	>0.08	<0.08	>0.08	<5	>0.08

a *Comparative Fit Index*,

b *Root Mean Square Error of Approximation*,

c *Tucker-Lewis Index*,

d *Chi- square/DF, and*

e *Incremental fit index*.

**Figure 3:**
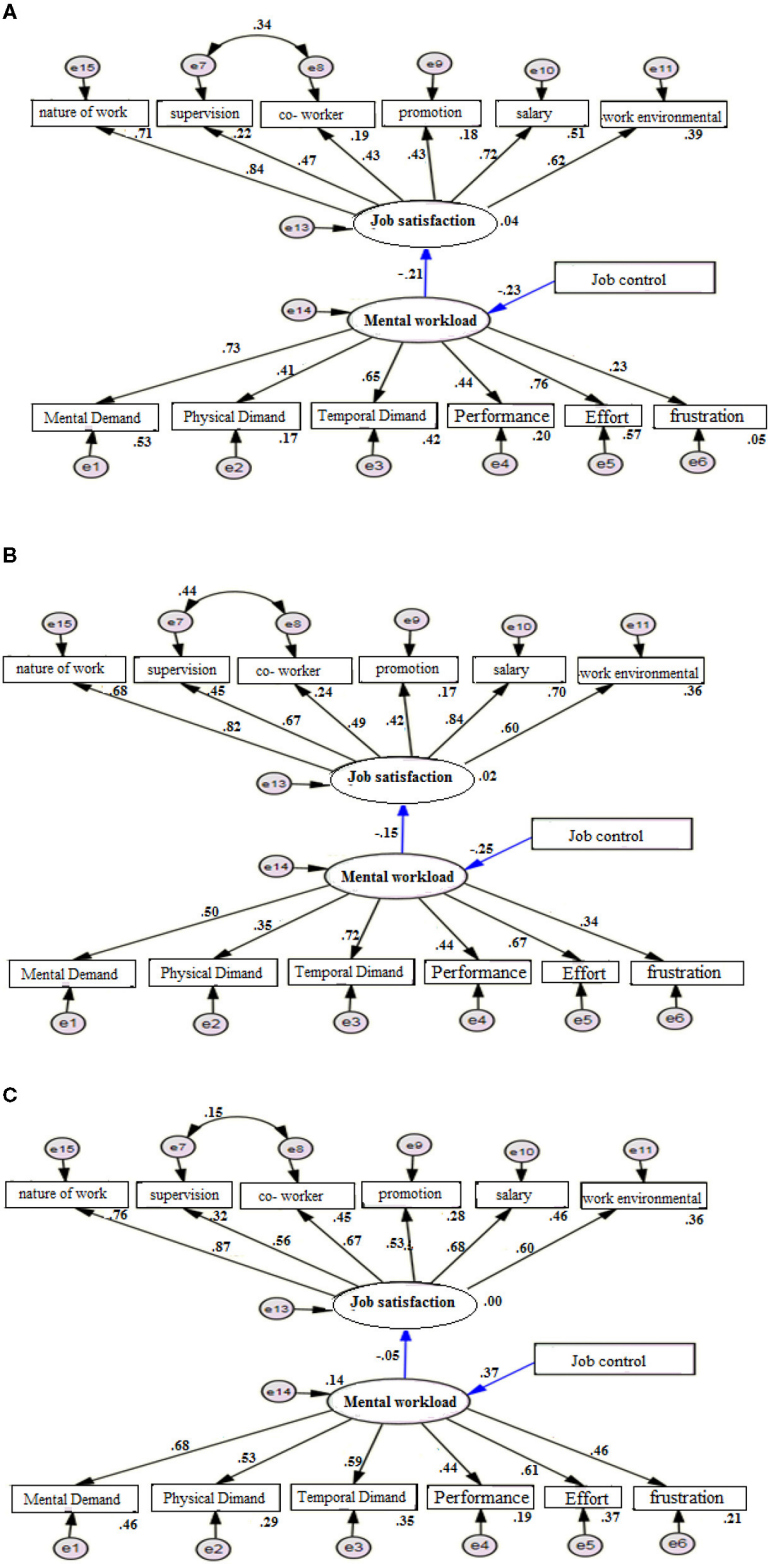
The model of relationship between variables of divided occupational groups; **(A)** nurses, **(B)** midwives, and **(C)** administrative staff.

The correlation between the workload and job satisfaction in the presence of the job control was separately investigated in the three job groups (Figure 3). In the designed models (2a−2c), the standard coefficient between the variables of workload and job satisfaction in the three groups of nursing, midwifery, and administrative workers were *r* = −0.21, *r* = −0.15, and *r* = −0.05, respectively (*P* < 0.001); while the standard coefficient of job control on the workload were *r* = −0.23, *r* = −0.25, and *r* = −0.375, respectively (*P* < 0.001). The goodness of fit index of the designed models for investigation of the relationship between workload and job satisfaction in the presence of the job control variable in the three job groups showed that the designed models have a good fit (Table 5).

**Table 5:** The relationship model between mental workload and job satisfaction by considering the job control variable by occupational groups.

**Job**	**CMIN/DF**	**IFI**	**TLI**	**CFI**	**RMSEA**	**RMR**
Nurse	1.646	0.923	0.902	0.921	0.059	0.030
Midwife	2.254	0.847	0.804	0.824	0.072	0.038
Administrative staff	2.186	0.846	0.804	0.824	0.078	0.044
Acceptable level	<5	>0.08	>0.08	>0.08	<0.08	<0.05
**Measure**	**Threshold**					
Chi-square/df (cmin/df)	<3 good; <5 sometimes permissible					
*P*-value for model	>0.05					
CFI	>0.95 great; >0.90 traditional; >0.80 sometimes permissible					
GFI	>0.95					
AGFI	>0.80					
SRMR	<0.09					
RMSEA	<0.05 good; 0.05–0.10 moderate; >0.10 bad					
PCLOSE	>0.05					

## Discussion

### The Present Situation of Mental Workload Job Satisfaction

In this study, the mean mental workload was high indicating that employees faced relatively high mental stress. The mean score of job satisfaction indicates the relative satisfaction of the individuals with conditions and work environment. Furthermore, the level of job control showed that the hospital workforce had a proper control in performing their duties. In consonance with the results, the level of mental workload in nurses was higher than in midwives and the administrative workers had the least level of workload. This trend is reversed for job satisfaction and job control, and the levels of job satisfaction and control among the administrative workers were higher than nurses and midwives. The activities demanding high concentration and attention create significantly great mental workload in the human operators to achieve a specific level of performance (42).

Other Studies have revealed a positive correlation between the job satisfaction and job performance of nurses and midwives (18, 19). The increase in work-hours is one of the causes of job dissatisfaction. It has a negative impact on the quality of services provided with the patient and decreases the patients' satisfaction with hospital services (20).

The level of job control between nurses and midwives was almost similar. The results indicated that by lowering the workload in the employees, more job satisfaction is observed, and they will have a better control over the assigned affairs. The difference in the amount of mental workload between nurses and midwives can be attributed to the work environment and more clients referred to nurses compared to midwives (6).

In addition, the clients, who referred to nurses, have predominantly acute and special situations and sometimes are overcrowded in the emergency department at the times of chain accidents and similar cases, which lead to more stress in nurses (40, 43). According to a study by Oliveira et al. nurses experienced a higher workload than other occupational groups, which was more related to poor working conditions and to a lack of job satisfaction (44). In a study conducted by Muhammadani et al. midwives were highly satisfied with organizational structure and rewards; while they were less satisfied with the work environment and job opportunities (45). In the study of Gouzou et al. the cause of low job satisfaction in nurses was related to rotational shifts in addition to workload (46).

Here, variables such as age and work experience had a significant relationship with mental workload. In the study of Wihardja et al., no significant relationship was observed between age, work experience, and mental workload of employees (47). They stated many other factors influence nurses in response to stimulation from their work. The length of working time can cause boredom and create job stress in person, however other factors inside or outside the nurse's capacity (48) also Studies showed psychological factors as one of risk factors in creating MSDs, in the initial phase of development, is important in workplaces (42).

In our study, job satisfaction in elderly healthcare workers was higher compared to younger people. The findings were consistent with the results studies of Carrillo-García et al. (49), and Alcaraz-Mor et al. (50). In the study of Singh et al., the most satisfied professionals were the youngest and the oldest (51); another study showed workers in the intermediate age range having the highest satisfaction levels (52). In this study, no significant relationship was found between age and work experience with job control. Since the job control is rooted in one's skill in doing the job and it can be independent of the individual age or work experience; thus, the job control is higher in the individuals with more skills.

### The Relationship Between Mental Workload and Healthcare Workers Job Satisfaction

This study investigated the relationships between mental workload and job satisfaction of the healthcare workers and then, the mediating role of the job control variable in this relationship evaluated. The results showed that there was a significant and inverse correlation between mental workload and job satisfaction. The findings are in agreement with the results of previous studies (44, 46). However, in the study of Oscar et al. no correlation was found between job satisfaction and mental workload, stating that mental workload scores associated with the practice of telemedicine were high and remained stable over time (16). According to Goetz et al. there was not any correlations between physical activities and job satisfaction in general physicians, and job satisfaction had a significant relationship with health indicators (53). Job satisfaction leads to improving performance (6). In this regard, management can play an important role in creating a suitable environment in the workplace (18). Studies have shown that job satisfaction depends more on organizational factors than individual factors (11, 54). Organizational factors can improve the quality of work life of employees and be associated with job satisfaction (15, 54). The workload can lead to aspects of burnout, including emotional exhaustion and depersonalization in healthcare workers (22). In addition, it causes a feeling of personal failure and involvement in employees (24, 25). That is if individuals have more skills and more control over their responsibilities, then they will have a higher job satisfaction.

In the face of staff shortages among nurses, which are intensifying due to the aging of society, it is necessary to diagnose factors that increase the stressfulness of work, so that effective actions to counteract them can be taken. Particular attention should be paid to young people, with less work experience and better education, as they are the most susceptible to the psychosocial burden and leave the profession the most often (55). Workload and job satisfaction of healthcare workers also can be effective in providing appropriate services with patients (16). On the other hand, job dissatisfaction and lack of balance among workload, ability, and individual limitations may affect the public health (29).

As mentioned before, the designed model was a representative of a significant and negative correlation between mental workload and job satisfaction, and despite the job control, as a moderating variable, the relationship between the two variables of workload and job satisfaction increased. As the job control increases, mental workload of individuals was somehow moderated and the following job satisfaction was raised. Portoghese et al., investigated the moderating role of the job control in the relationship between workload and burnout among healthcare workers. The results indicate that a low job control can strengthen the positive and significant correlation between workload and burnout (31); which is consistent with our study. In the study of Adibi et al., the role of job control was observed in the relationship between positive and negative behaviors of employees (41). With respect to the demand-job control theory, the stress in work environments occurs for people when the level of job demands is high and in contrast, job control is low (32). In contrast, Martinussen et al. showed that job control beyond the job demand has a significant predictive power for burnout Therefore, job control as a potential moderator variable can play a good role in patterns such as demand-control-behavioral consequences (33).

Among the aspects of mental workload, the highest score was related to effort. Moreover, among the variables of job satisfaction, the highest score was related to the nature of work while the results of studies on nurses showed that the highest score among the workload aspects was related to the time pressure (56). The results of the other studies showed that the performance pressure was lower compared to the other aspects of workload (17). Taheri et al., showed that in addition to physical pressure, the nurses are subjected to other complications such as the pressure of time, a lack of control over the work pace, and psychological need that play an influential role in needle stick injuries (57). Based on the designed model, the relationship between the variables of mental workload and job satisfaction was more robust in nurses than in midwives and administrative workers. It can be due to the high level of mental workload and the variety of the nurses' responsibilities. However, the time pressure and mental workload were lower in administrative workers than those in the treatment department, and consequently, this relationship was not much strong despite the desirability of the designed model.

Improving organizational support can represent a target for both managers and workers who want to mitigate the negative consequences of stress, both in economic and health terms (58).

## Limitations

The study was performed using questionnaires, which can bias the results. Physicians did not participate in this study, although they can play a major role in hospital settings. Moreover, the study was restricted to the several state hospitals. Further research may look at the outcomes of private hospitals and compare them to the findings of this report.

## Conclusion

The mental workload in healthcare was high and the results showed that job satisfaction diminished by an increase in mental workload. Job control can play an important role in improving the working conditions of healthcare workers and in increasing the job satisfaction. It is important to develop organizational management practices that enable job control; therefore, the management strategies should be adopted to diminish the workload pressure and increase the job control in order to enhance the job satisfaction among healthcare workers. In this regard, managers can temporarily reduce the workload by providing a supple program such as a floating workforce.

## Data Availability Statement

The raw data supporting the conclusions of this article will be made available by the authors, with permission from Ethics Committee of SFMS.

## Author Contributions

MF-A and FR: study conception and design. AB-P and ZM: data collection. AJ and MF-A: analysis and interpretation of results. GT-B, FR, MF-A, and AB-P: draft manuscript preparation. All authors reviewed the results and approved the final version of the manuscript.

## Conflict of Interest

The authors declare that the research was conducted in the absence of any commercial or financial relationships that could be construed as a potential conflict of interest.

## Publisher's Note

All claims expressed in this article are solely those of the authors and do not necessarily represent those of their affiliated organizations, or those of the publisher, the editors and the reviewers. Any product that may be evaluated in this article, or claim that may be made by its manufacturer, is not guaranteed or endorsed by the publisher.
